# Beyond the Situation: Hanging Out with Peers now is Associated with Short-Term Mindsets Later

**DOI:** 10.1007/s40865-024-00249-2

**Published:** 2024-02-17

**Authors:** Sebastian L. Kübel, Jessica R. Deitzer, Willem E. Frankenhuis, Denis Ribeaud, Manuel P. Eisner, Jean-Louis van Gelder

**Affiliations:** 1 Max Planck Institute for the Study of Crime, Security and Law, Günterstalstraße 73, D-79100 Freiburg im Breisgau, Germany; 2 Institute of Education and Child Studies, Faculty of Social and Behavioral Sciences, Leiden University, Leiden, The Netherlands; 3 Evolutionary and Population Biology, Institute for Biodiversity and Ecosystem Dynamics, University of Amsterdam, Amsterdam, The Netherlands; 4 Department of Psychology, Utrecht University, Utrecht, The Netherlands; 5 Jacobs Center for Productive Youth Development, University of Zurich, Zurich, Switzerland; 6 Violence Research Centre, Institute of Criminology, University of Cambridge, Cambridge, UK

**Keywords:** Unstructured Socializing, Peer Influence, Short-term Mindsets, Socialization, Fixed-effects Models, Longitudinal

## Abstract

It is well-established that unstructured unsupervised socializing with peers (UUS) motivates deviance while in that specific context. In this article, we extend this situational view by arguing that repeated UUS may also gradually shape adolescents’ norms and decision making beyond the situation. Specifically, we argue that UUS promotes short-term mindsets, i.e., an increased focus on present rewards at the expense of considering future consequences. We test this hypothesis with fixed-effects models, using longitudinal data from a representative sample of 1,675 adolescents from Zurich, Switzerland. Consistent with our preregistered predictions, more frequent UUS is associated with increased short-term mindsets. Thus, our finding suggests that the effects of UUS on later deviance might be driven by becoming more present-oriented. This link offers new insights into the developmental pathways toward adolescent delinquency and offers a potential target for intervention.

**Supplementary Information:**

The online version contains supplementary material available at 10.1007/s40865-024-00249-2.

## Introduction

Adolescents benefit from hanging out with peers (Flynn et al., 2023; Larson et al., 2006), but such interactions also put them at greater risk for detrimental outcomes (Hoeben & Weerman, 2014). Spending time with peers without a predefined purpose and adult supervision is commonly referred to as unstructured unsupervised socializing (UUS; Osgood et al., 1996). Negative outcomes of UUS include victimization, smoking, substance use, early sexual intercourse, low school achievement, dangerous driving, and delinquency (Badura et al., 2018; Chen, 2009; Dong et al., 2020; Osgood et al., 1996). The link between UUS and deviance holds across research methodologies, sample characteristics, and types of deviance (for an overview, see Hoeben et al., 2016).

It has been suggested that the effects of UUS on deviance are due to features of the situation rather than socialization (Osgood et al., 1996; see Hoeben et al., 2016, for review). That is, UUS is thought to motivate deviance only while peers are present; once adolescents leave the situation, they also leave the risk behind (Osgood et al., 1996). Situational peer effects contrast with socialization effects, whereby peers are viewed as causing *sustained changes* in attitudes by transmitting norms and preferences that either promote deviance and delinquency or not (Hoeben et al., 2016; Hoeben & Thomas, 2019; Pratt et al., 2010; Sutherland, 1947). While UUS was initially conceived as a strictly situational explanation, scholars are beginning to acknowledge the socialization potential for UUS (Hoeben & Thomas, 2019; Hoeben & Weerman, 2016).

In this article, we advocate for this theoretical shift and hypothesize that frequent UUS increases short-term mindsets in adolescents, and that it does so also *beyond the immediate situation*. We use ‘short-term mindsets’ as an umbrella term referring to concepts characterized by an increased focus on current versus future outcomes, including, but not limited to, impulsivity, sensation-seeking and low future orientation (Kübel et al., 2023; van Gelder et al., 2020).^1^ We test our hypothesis by assessing whether individuals exhibit increased short-term mindsets after periods in which they have spent more time in UUS.

All of the aforementioned detrimental outcomes associated with UUS are also linked to short-term mindsets (de Ridder et al., 2012; Forrest et al., 2019; Pratt et al., 2014; van Gelder et al., 2018; Gelder et al., 2020; Vazsonyi et al., 2017). Research also demonstrates an increased focus on the present while with peers (e.g., Gilman et al., 2014; O’Brien et al., 2011). If UUS reinforces short-term mindsets beyond the situation, this may explain the link between UUS and later deviance (Hoeben et al., 2016). Such a finding would support the perspective that effects of UUS are not exclusively situational (Hoeben & Thomas, 2019).

### Current Theory and Research on UUS

Currently, UUS is theorized to be a criminogenic context that provides opportunities and rewards for deviance (Osgood et al., 1996). The lack of adult supervision reduces both social control and the chance of apprehension, while the lack of structure may facilitate deviant opportunities. The presence of peers can motivate deviant behavior *passively* by modeling the behavior or affecting perceived social costs and rewards (Hoeben & Thomas, 2019). That is, when peers are present, social rewards are particularly salient (Foulkes & Blakemore, 2016; Smith et al., 2013, 2015, 2018; Steinberg, 2008) and status gains and the prevention of status losses among peers are major incentives for engaging in deviance (Thomas & Nguyen, 2022; see also Falk et al., 2014). Peers may also *actively* influence decision making by instigating or encouraging deviance, exerting pressure, or demanding conformity (Hoeben & Thomas, 2019; Hoeben & Weerman, 2016).

The criminogenic effect of UUS does not depend on the presence of peers with a delinquent propensity (Haynie & Osgood, 2005). Rather, the context itself appears to evoke the increased motivation for deviance. UUS is associated with increased delinquency in adolescents even after accounting for the delinquency of their peers (Gerstner & Oberwittler, 2018; Haynie & Osgood, 2005; Svensson & Oberwittler, 2010; Weerman et al., 2015). Put simply, being with (any) peers in UUS is associated with more delinquency.

### Peer Influence on Short-Term Mindsets

Here, we propose that UUS promotes deviance through increasing short-term mindsets. The concept of short-term mindsets is similar to (low) self-control, but is informed by recent theory and findings (see Burt, 2020, for review; see Kübel et al., 2023; van Gelder et al., 2020, for further discussion). First, short-term mindsets narrows in on intertemporal decision-making tendencies. It subsumes only self-control concepts that describe a tendency to prioritize the present and disregard long-term costs: impulsivity and sensation-seeking (Gottfredson & Hirschi, 1990, p. 177, 2019, p. 4; van Gelder et al., 2020). However, the definition also covers other concepts not included in self-control, such as (low) future orientation, future discounting, and inability to delay gratification. Second, we analyze different indicators of short-term mindsets separately, rather than as components of a higher-order construct, as is the case with self-control. This approach takes into account that these indicators are interrelated, though distinct, concepts with different neurological roots and developmental trajectories (Forrest et al., 2019; Steinberg et al., 2008, 2009). Third, and importantly, studies have shown considerable between- and within-individual change in self-control, also beyond childhood (Burt et al., 2006, 2014; Meldrum et al., 2012). The term ‘mindsets’ serves to emphasize this variability over time. Recent research reveals how environmental and social factors may influence short-term mindsets. For example, harsh parenting practices, victimization, future uncertainty, sanctions, and poverty affect the focus on present outcomes (Frankenhuis et al., 2016; Kübel et al., 2023; Pepper & Nettle, 2017; van Gelder et al., 2018; Gelder et al., 2020; Wojciechowski, 2022). Short-term mindsets are thus a response to environmental and social factors, including peers, even if they are influenced by dispositional factors as well (Fenneman et al., 2022).

The empirical literature has identified two different processes through which peers increase short-term mindsets situationally. The first is their *mere presence*. fMRI studies find that the presence of peers provokes greater activation of reward-related brain regions in adolescents, which in turn predicts higher risk-taking than when alone (Chein et al., 2011; Smith et al., 2015, 2018). Adolescents discount the future more when peers are present (O’Brien et al., 2011). This heightened preference for immediate rewards even shows up when participants are made to believe that an anonymous peer, whom they do not meet or see, is observing them (Weigard et al., 2014). Another study replicates the peer effects on delay discounting and on risk-taking in college students; however, when a single adult is present in the group, replacing one of the peers, discounting and risk-taking are comparable to when the tasks are completed alone (Silva et al., 2016). These findings suggest that the mere presence of peers can increase short-term mindsets.

Second, peers can increase short-term mindsets in a situation by conveying information about their norms and preferences. One way of doing so is through *passive modeling*. Experimental studies indicate that people become more present-oriented and take more risks after observing present-oriented behavior by peers. For example, seeing other people make more impulsive choices in a delay discounting task influences young adults’ own impulsive choices (Gilman et al., 2014). Also, male adolescents are more likely to make risky choices in a gambling task after observing peers do the same (Reiter et al., 2019; see also Suzuki et al., 2016). This adjustment might be deliberate: adolescents who are better at learning to predict their peer’s choices also display more conformity to their peers’ choices (Reiter et al., 2019).

Another way peers convey norms and preferences is by *actively* encouraging or verbally communicating attitudes favorable to present-oriented behavior. For example, online advice by a peer that encourages risk-taking makes adolescents more risk-taking in a gambling task (van Hoorn et al., 2017). In a simulated driving experiment, male adolescents are more risk-taking with peer passengers compared to when driving alone; moreover, this effect only emerges with risk-accepting passengers, not when passengers are explicitly risk-averse (Simons-Morton et al., 2014). When risk-taking peers give advice, adolescents have more accidents in a simulated driving task, compared to when these peers are merely present (Centifanti et al., 2016). Thus, peer effects on situationally driven short-term mindsets become stronger in the context of normative information.

However, longitudinal studies suggest that the effects of exposure to present-oriented peers are not limited to situational influence but may also be enduring. The impulsivity of peers is associated with increases in one’s own level of impulsivity over time; crucially, this effect holds after accounting for the fact that adolescents prefer to select friends with a level of impulsivity similar to one’s own (Ragan et al., 2022). Being around deviant peers and peers with low self-control is also associated with later decreases in self-control (e.g., Burt et al., 2006; Huijsmans et al., 2021; Jennings et al., 2013; Meldrum et al., 2012; Meldrum & Hay, 2012).

Altogether, this evidence supports the claim that, while socializing, adolescents learn about peers’ norms and preferences, including their short-term mindsets. In turn, friendship with present-oriented peers (which likely means frequent socializing) is associated with later increases in one’s own short-term mindsets. What needs to be tested is whether the situational effect specifically of UUS on short-term mindsets translates into a change beyond the situation.^2^

### UUS Effects beyond the Situation

Recently, scholars have proposed that the effects of UUS on deviance may reach beyond the situation and also have sustained effects as UUS experiences accumulate. Specifically, Hoeben and Thomas (2019) suggest that repeated UUS may lead adolescents to learn from this “chain of situations,” as they learn from any other experiences. They argue that “socialization by peers is the result of individuals’ cumulative experience with exposure to situational peer influence. Just as lives are built one day at a time, perceptions and preferences develop gradually from responses to daily social interactions” (p. 766).^3^ Consistent with this idea, socialization may partially account for the link between UUS and later delinquency. In a longitudinal study of Dutch adolescents, time spent in UUS was associated with greater exposure to deviant peers, higher tolerance for substance use and offending, increased perceived opportunities for delinquency, and elevated peer reinforcement for deviant behaviors; all of these variables partially mediated the association of UUS and later delinquent behavior (Hoeben & Weerman, 2016).

There are two important reasons why non-situational effects of repeated exposure to UUS deserve further consideration. First, most of the existing tests of UUS effects on deviance have relied on data that did not distinguish between deviance within and outside of UUS contexts (for exceptions using situational data, see Bernasco et al., 2013; Chrysoulakis et al., 2022; de Jong et al., 2020; Wikström et al., 2012). Most studies measure UUS as *how often* adolescents are in UUS during a specified period of time (a month, a year, etc.). As a consequence, these studies have investigated whether UUS is associated with more deviance in general, not specifically within that situation. The observed effect of frequent UUS on deviance could thus be (partly) due to deviance that happens after leaving the situation, which may reflect socialization (for review, see Hoeben et al., 2016). In sum, most prior studies cannot distinguish between situational and socialization effects. Second, prior studies suggest that adolescents make more present-oriented decisions even when their peers are not directly present (e.g., Gilman et al., 2014; Reiter et al., 2019). Furthermore, short-term mindsets appear to increase after periods of frequent exposure to friends who exhibit such mindsets (e.g., Meldrum et al., 2012; Ragan et al., 2022). We propose that the more often adolescents are in situations of UUS, the more likely they are to prioritize the present over the future in general.

We are aware of only a single study linking UUS and (low) self-control, a concept akin to short-term mindsets. In a between-subjects design, more UUS was associated with lower self-control, and socialization mediated this relationship (Archer  & Flexon, 2021). However, the cross-sectional data in this study precludes inferences about individual change over time (Kraemer et al., 2000). In addition, the predominantly male (86%) sample of adolescent serious offenders limited the generalizability of the findings. Our study addresses both of these issues by using longitudinal data from a representative sample of adolescents.

### The Current Study

We investigate whether more frequent UUS is associated with increased short-term mindsets, using impulsivity, sensation-seeking, and (lack of) future orientation as indicators of short-term mindsets. Specifically, we examine whether UUS is associated with increased short-term mindsets not only during UUS, but also beyond the current situation. We investigate the following predictions:

(1) Within persons, increases in UUS will be associated with increased impulsivity, net of time-varying controls.

(2) Within persons, increases in UUS will be associated with increased sensation-seeking, net of time-varying controls.

(3) Within persons, increases in UUS will be associated with decreased future orientation, net of time-varying controls.

## Methods

### Participants

We use longitudinal data of a representative sample of *N* = 1,675 adolescents from the Zurich Project on the Social Development from Childhood to Adulthood^4^ (z-proso; Ribeaud et al., 2022). Caregivers gave informed consent for the first four waves and passive consent thereafter until age 17. Participants provided informed consent from wave 5 onwards. The adolescents completed the questionnaires in classrooms after school and received financial compensation. The Ethics Committee of the Faculty of Arts and Social Sciences of the University of Zurich approved the study^5^.

We used data from waves 4–8 of the z-proso project. These waves correspond to ages 11 to 20 of the adolescent cohort (see Table 1). On August 2, 2022, before data analyses, we preregistered our predictions, data processing, and statistical analyses^6^. We published the data, metadata, and analysis scripts on a public repository^7^.

**Table 1:** Description of the waves of the z-proso project

wave	assessment year	n (% of study sample = 1,675)	mean age (SD)
4	2009	1,148 (68.53%)	11.33 (0.37)
5	2011	1,366 (81.55%)	13.67 (0.36)
6	2013	1,447 (86.39%)	15.44 (0.36)
7	2015	1,306 (77.97%)	17.45 (0.37)
8	2018	1,180 (70.45%)	20.58 (0.38)

### Measures

We present the full list of items of all measures in Appendix A, internal consistencies of the scales in Appendix B, and descriptive statistics for all scales and waves in Appendix C.

### Unstructured Unsupervised Socializing (UUS)

We use the mean of five items^8^ measuring the frequency of UUS on a six-point Likert scale from 1 = never to 6 = (almost) every day (e.g., “How often do you hang around with friends in a park, in the train station, or in a shopping mall, and have fun, in the evening”). The scale is based on Wetzels et al. (2001).

### Short-Term Mindsets

We use three separate indicators of short-term mindsets. We measure impulsivity and sensation-seeking with two items each. Both are derived from the shortened version of the Grasmick et al. (1993) self-control scale. A sample item of impulsivity is “I often do whatever brings me pleasure here and now, even at the cost of some distant goal,“ and for sensation-seeking, “Excitement and adventure are more important to me than security.” We measure future orientation with three items assessing the adolescents’ long-term educational aspirations (e.g., “I try hard at school to have a good job later in life”); this scale was created and psychometrically pretested by the z-proso Research Team. The scores range from 1 (false) to 4 (true) for all items. We use the mean of the respective items for each of these three indicators.

### Control Variables

As we discuss below, fixed-effects models control for time-stable factors of individual differences. Additionally, we included time-varying control variables from the same waves. We selected potential confounding variables with commonly demonstrated associations with UUS and short-term mindsets (for more information, see Appendix A). Aside from participant age, we control for the following variables:

#### Offending

A scale adapted from Wetzels et al. (2001) assessed whether the respondent committed any of 14 types of offenses in the past 12 months (e.g., “In the past 12 months, have you ever forcibly taken money or things from someone?”; 0 = no; 1 = yes). We use item response theory^9^ for these dichotomous items at each wave (Osgood et al., 2002).^10^ Based on the item scores, we derive individual values (theta levels *θ*) on a latent trait dimension reflecting criminal propensity at each wave (Osgood et al., 2002).

#### Affiliations with Delinquent Peers

Participants nominated up to two friends and reported whether these had engaged in either assault and/or shoplifting in the past year (e.g., “In the last year, has he/she purposely hit or kicked another adolescent and injured them in the process?”). We compute a variable of delinquent peers, representing the proportion of friends that exhibited any of these two delinquent acts (either 0, 0.5, or 1; Haynie, 2002).

#### Parental Monitoring

The measure of parental monitoring consists of two subscales. The first subscale measures parental supervision as the mean of four items (e.g., “When you go out in your free time, your parents ask you where you are going”). The second subscale measures adolescent disclosure with the mean (reverse coded) scores of three items (e.g., “You keep secret from your parents what you do in the evenings and at the weekends”). 11 All items are rated on a four-point Likert scale (ranging from 1 = never to 4 = often/always). We use the mean of the respective items for both subscales. All items are based on the Alabama Parenting Questionnaire (Shelton et al., 1996) and the Parenting Scale (Wetzels et al., 2001).

#### Parental Involvement

Six items measure parental involvement, scored on a four-point Likert scale (e.g.; “When you have a problem, you can talk to your parents about it;” 1 = never; 4 = often/always).^12^ We use the mean of these six items. All items are based on the Alabama Parenting Questionnaire (Shelton et al., 1996) and the Parenting Scale (Wetzels et al., 2001).

### Statistical Analyses

We use fixed-effects models to estimate the association between changes in UUS (independent variable) and changes in short-term mindsets (the dependent variable) across waves. These models quantify deviations from peoples’ average scores on the variable, eliminating unmeasured time-stable heterogeneity (Allison, 2009). Thus, our models compare times when a person was more engaged in UUS to times when the person spent less time in this context (Allison, 2009; Bernasco et al., 2013). Fixed-effects models are considered “the most rigorous test for intra-individual effects” (Weulen Kranenbarg et al., 2018, p. 352).

We run three sets of fixed-effects regression models with Huber/White/sandwich robust standard errors (Huber, 1967; White, 1980). Each set consists of three models, one for each of the indicators of short-term mindsets: impulsivity, sensation-seeking, and (low) future orientation. The models for impulsivity and sensation-seeking include waves 4–8. The models for future orientation involve waves 5–7, as this measure is not available at waves 4 and 8. The first set of models estimates the within-person association between UUS and each of the three indicators of short-term mindsets across all available waves, without control variables. In the second set, we control for variations in offending, delinquent peer affiliations, and age. The third set additionally includes the parenting control variables. As these parenting controls are not available at wave 8, this third set incorporates only waves 4–7. Note that all time-varying control variables could also potentially mediate the relationship of UUS with short-term mindsets. Thus, the actual effects of UUS on short-term mindsets may be underestimated with these controls included.

In addition, we also run three sets of exploratory models including an interaction of UUS and delinquent peers on the short-term mindsets indicators. Interaction effects would indicate that the relationship between UUS and short-term mindsets is affected by whether the individual hangs out with delinquent peers. Prior research finds mixed support for such interaction effects on delinquency (Gerstner & Oberwittler, 2018; Haynie & Osgood, 2005; Svensson & Oberwittler, 2010).

We also run three exploratory models (one bivariate, two with controls) where short-term mindsets is captured through a single latent variable. While research shows they are distinct concepts (e.g., Steinberg et al., 2008, 2009), they all involve the prioritiation of the present, which justifies exploring an alternative analytic approach that examines their shared variance. As such, we estimate latent variable scores for each individual based on the scores on the items of all three indicators.

We took all measures of UUS, short-term mindsets, and the control variables from the same wave. However, assessment of the controls (excluding age) and the frequency of time spent in UUS requires a retrospective evaluation (e.g., offending in the past 12 months). In contrast, the measures of short-term mindsets were worded in the present tense. Thus, the temporal order of these measures allows for testing our hypotheses about UUS effects on *later* short-term mindsets.

For each of the models, we excluded observations (participants/year) from the analyses when they had missing data on any of the variables (listwise deletion). We conducted all confirmatory analyses in StataMP 16 using two-tailed null hypothesis significance tests (*α* = 0.05).

## Results

### Bivariate Correlations

There is a consistent association between UUS and all three indicators of short-term mindsets (*p* < .001 for all waves). The strength of these correlations with UUS may be evaluated as medium positive for impulsivity, as medium to strong positive for sensation-seeking, and as weak to medium negative for future orientation (Gignac & Szodorai, 2016). We provide the bivariate correlations (Spearman’s rho) in Appendix D.

### Bivariate Fixed-Effects Models Excluding Control Variables

Consistent with our preregistered predictions, the bivariate fixed-effects models showed that within-individual increases in the frequency of spending time in UUS are significantly associated with increases in all indicators of short-term mindsets over time (see Table 2). UUS was positively associated with impulsivity (*b* = 0.141, *t*(1519) = 13.78, *p* < .001) and sensation-seeking (*b* = 0.182, *t*(1519) = 16.53, *p* < .001), and negatively associated with future orientation (*b* = -0.073, *t*(1479) = -5.06, *p* < .001).

**Table 2:** Results of the bivariate fixed-effects models

	impulsivity	sensation-seeking	future orientation
	*b* (SE)	*b* (SE)	*b* (SE)
**(a) Model parameters**			
UUS	0.141*** (0.010)	0.182*** (0.011)	-0.073*** (0.014)
constant	1.855*** (0.028)	1.563*** (0.031)	3.399*** (0.042)
**(b) Model characteristics**			
*n* (unique individuals)	1,521	1,521	1,481
*N* (person-waves)	6,388	6,386	3,995
rho	0.355	0.480	0.486
model	*F*(1,1520) = 189.89***	*F*(1,1520) = 273.38***	*F*(1,1480) = 25.65***

Note. The models for impulsivity and sensation-seeking involve waves 4–8, whereas the model for future orientation comprises only waves 5–7 because this scale was not available in the other waves

* *p* < .05; ** *p* < .01; ***p < .001. UUS = unstructured unsupervised socializing. *b* = unstandardized coefficient. SE = robust Huber/White/sandwich standard error. OR = odd’s ratio = exp(*b*), i.e. the predicted change in odds for a unit increase in the predictor. rho = fraction of variance due to fixed effects. The model *F*-statistic tests whether all fixed effects are zero

### Fixed-Effects Models Including Control Variables

The regression coefficients slightly decrease in magnitude after inclusion of the controls (see Table 3), but UUS remains positively associated with impulsivity (*b* = 0.127, *t*(1511) = 10.83, *p* < .001) and sensation-seeking (*b* = 0.168, *t*(1511) = 13.63, *p* < .001), and negatively associated with future orientation (*b* = -0.044, *t*(1462) = -2.73, *p* = .006).

**Table 3:** Results of the bivariate fixed-effects models including control variables, except for the parenting variables

	impulsivity	sensation-seeking	future orientation	
	*b* (SE)	*b* (SE)	*b* (SE)	
**(a) Model parameters**				
UUS	0.127*** (0.012)	0.168*** (0.012)	-0.044** (0.016)	
*control variables*:				
age	0.006† (0.003)	-0.002 (0.003)	-0.021*** (0.006)	
offending	0.059*** (0.017)	0.148*** (0.018)	-0.103*** (0.021)	
delinquent peers	0.072* (0.035)	0.164*** (0.035)	-0.085* (0.041)	
constant	1.791*** (0.047)	1.603*** (0.048)	3.651*** (0.090)	
**(b) Model characteristics**				
*n* (unique individuals)	1,516	1,516	1,467	
*N* (person-waves)	5,872	5,871	3,720	
rho	0.365	0.462	0.528	
model	*F*(4,1515) = 50.38***	*F*(4,1515) = 91.87***	*F*(4,1466) = 18.70***	

Note. The models for impulsivity and sensation-seeking involve waves 4–8, whereas the model for future orientation comprises only waves 5–7 because this scale was not available in the other waves

† *p* < .10; * *p* < .05; ** *p* < .01; ***p < .001. UUS = unstructured unsupervised socializing. *b* = unstandardized coefficient. SE = robust Huber/White/sandwich standard error. rho = fraction of variance due to fixed effects. The model *F*-statistic tests whether all fixed effects are zero

Additionally including the three parenting controls further decreases the regression estimates (see Table 4), but the positive associations of UUS with impulsivity (*b* = 0.093, *t*(1506) = 6.30, *p* < .001) and sensation-seeking (*b* = 0.126, *t*(1506) = 8.68, *p* < .001) remain significant. In the model for future orientation, the additional control variables render the association between UUS and future orientation nonsignificant (*b* = -0.023, *t*(1459) = -1.41, *p* = .159).

**Table 4:** Results of the bivariate fixed-effects models including all control variables

	impulsivity	sensation-seeking	future orientation
	*b* (SE)	*b* (SE)	*b* (SE)
**(a) Model parameters**			
UUS	0.093*** (0.147)	0.126*** (0.015)	-0.023 (0.016)
*control variables*:			
age	0.028*** (0.005)	0.005 (0.005)	-0.011† (0.006)
offending	0.040* (0.020)	0.113*** (0.021)	-0.085*** (0.020)
parental supervision	− 0.049* (0.020)	-0.008 (0.022)	0.016 (0.022)
adolescent disclosure	-0.099 (0.022)	-0.159*** (0.021)	0.094*** (0.023)
parental involvement	-0.041 (0.027)	-0.147*** (0.026)	0.152*** (0.027)
delinquent peers	0.059 (0.037)	0.146*** (0.040)	-0.058 (0.041)
constant	2.183*** (0.150)	2.618*** (0.152)	2.644*** (0.167)
**(b) Model characteristics**			
*n* (unique individuals)	1,514	1,514	1,467
*N* (person-waves)	4,834	4,833	3,714
rho	0.384	0.476	0.517
model	*F*(7,1513) = 46.35***	*F*(7,1513) = 60.48***	*F*(7,1466) = 19.93***

Note. The parenting control variables (parental supervision, adolescent disclosure, parental involvement) were only available from waves 4–7. Therefore, models for impulsivity and sensation-seeking involve waves 4–7. The model for future orientation comprises only waves 5–7 because this scale was not available in wave 4

† *p* < .10; * *p* < .05; ** *p* < .01; ***p < .001. UUS = unstructured unsupervised socializing. *b* = unstandardized coefficient. SE = robust Huber/White/sandwich standard error. rho = fraction of variance due to fixed effects. The model *F*-statistic tests whether all fixed effects are zero

### Exploratory Analyses

As exploratory analyses, we ran all models again, including the interaction of UUS with the delinquent peers measure. Results indicate that the significance of UUS’ associations with short-term mindsets remained similar as in the confirmatory models. Delinquent peers were significantly, albeit weaker, associated with short-term mindsets in almost all models. There was no interaction effect between UUS and delinquent peers in any of the models (see Table 5, for an overview, and Appendix E for the detailed results).

**Table 5:** Overview of the results of the fixed-effects models including the interaction of delinquent peers and UUS

	impulsivity			sensation-seeking			future orientation		
number of controls	none	reduced	full	none	reduced	full	none	reduced	full
UUS	***	***	***	***	***	***	***	**	n.s.
delinquent peers	**	*	†	***	***	***	**	*	†
UUS x delinquent peers	n.s.	n.s.	n.s.	n.s.	n.s.	n.s.	n.s.	n.s.	†

Note. none = model without control variables; reduced = model with offending and age control variables; full = model with all control variables, including the three parenting controls. This full model with all controls involves waves 4–7 (instead of waves 4–8) for impulsivity and sensation-seeking. All models with future orientation involve waves 5–7

† *p* < .10; * *p* < .05; ** *p* < .01; ***p < .001

We also ran the fixed-effect models with a factor of short-term mindsets that included all seven items of impulsivity, sensation-seeking and future orientation (standardized factor loadings were λ ≥ |0.35| for all items at all waves). There was a significant association between UUS and short-term mindsets (*b* = 0.153, *t*(1517) = 12.16, *p* < .001), which remained after the inclusion of all control variables (*b* = 0.114, *t*(1503) = 6.90, *p* < .001; see Appendix F).

## Discussion

We find evidence for our hypotheses that UUS has non-situational effects on short-term mindsets. Specifically, more time spent in UUS is associated with later increases in adolescents’ levels of impulsivity and sensation-seeking. UUS is also associated with later decreases in future orientation, but to a somewhat lesser extent. These findings suggest socialization effects of frequent involvement in UUS that extend beyond the immediate situation—as proposed by Hoeben and Weerman (2016).

Our study answers McGloin and Thomas’ (2019) call for research that contributes to understanding *how* peers influence behavior. Socialization and situational peer influence are commonly understood as two distinct mechanisms of peer influence (McGloin & Thomas, 2019). Delinquent peers are viewed a socializing force that shapes internalized norms and preferences through repeat modeling behavior and reinforcing of certain behavior. In contrast, the contextual features of UUS are seen as motivating deviance only in the immediate situation while peers are present (Hoeben et al., 2016; Osgood et al., 1996). Here, we soften this theoretical distinction by demonstrating how frequency of UUS can be related to changes in one’s preferences, specifically, their short-term mindsets. In doing so, we build upon the argument that repeated exposure to UUS can also be a cumulative source of socialization, shaping adolescent norms and preferences (Hoeben & Thomas, 2019).

We further argue that effects of UUS and peer delinquency are more intertwined than often presented (Hoeben et al., 2016). Frequent UUS may hold sustained effects in a similar way to that of delinquent peers (Hoeben & Thomas, 2019). Repeated UUS likely socializes adolescents in the (deviant) norms, preferences, and behaviors their peers are exhibiting in that setting (Hoeben & Weerman, 2016). Our findings are consistent with this claim. Additionally, UUS is linked to exposure to deviant peers (Archer et al., 2022; Boman, 2013; Hoeben & Weerman, 2016; Wong, 2005). Adolescents are especially likely to co-offend in peer groups (Warr, 2002), and co-offending may strengthen processes of normative criminogenic influence (Defoe et al., 2021). This makes the effect of UUS and of delinquent peers difficult to disentangle; both imply repeated exposure to others who either generally espouse deviant behaviors, or demonstrate them in UUS context.

However, our explanatory analyses did not provide evidence for an interaction between UUS and having delinquent peers on short-term mindsets. Rather, UUS and delinquent peers both seemed to hold independent effects. UUS may increase short-term mindsets irrespective of whether they have delinquent peers – aligning with some prior findings (Haynie & Osgood, 2005). Some prior research did find interactions, stating both that the effect of UUS on delinquency is dependent on peers’ delinquency, and that the effect of delinquent peers depends on the amount of time spent in UUS (Gerstner & Oberwittler, 2018; Svensson & Oberwittler, 2010).

Enduring increases in short-term mindsets may account for (part of) the link between UUS and delinquency or other maladaptive outcomes in previous longitudinal studies (e.g., Maimon & Browning, 2010). As noted, both UUS and short-term mindsets are linked with low school achievement, substance use, victimization, and delinquency, among others (Badura et al., 2018; de Ridder et al., 2012; Dong et al., 2020; Osgood et al., 1996; Pratt et al., 2014; Vazsonyi et al., 2017). Future research could examine the extent to which changes in short-term mindsets mediate the relationships between UUS and such detrimental outcomes.

Our findings allow for the possibility of a reciprocal relationship between UUS and short-term mindsets. Previous studies suggest that short-term mindsets are associated with greater involvement in UUS (Janssen et al., 2018; Maimon & Browning, 2010; Müller et al., 2013; Qu et al., 2021). It is plausible that people with higher short-term mindsets are more likely to select into risk-conducive contexts, such as UUS. Our findings suggest that the reverse may be true as well. That is, UUS might increase short-term mindsets, creating a feedback loop from risk-conducive contexts, such as UUS, to short-term mindsets, to UUS, and so forth. Future work could simultaneously demonstrate both arrows of this feedback loop over time, and explore which factors and processes are able to ‘break’ such a cycle.

Anticipating studies of feedback loops, we may ask: why do adolescents not become more and more present-focused as they age and time spent socializing with peers grows? One explanation is that adolescents naturally become adults and transition into new roles (Moffitt, 1993). Among these transitions are school completion, romantic partnership or marriage, and employment (see the idea of ‘turning points’ by Sampson & Laub, 1993). Preferences for impulsive and risk-taking behavior may decrease accordingly over time. Both existing and new peers may increasingly value other, more conventional status indicators (Matza, 1964).

Another explanation is that the salience of peer influence decreases over the course of adolescence. Specifically, susceptibility peaks in mid-adolescence, and decreases from age 14–15 onwards (see Laursen & Veenstra, 2021, for review; Steinberg & Monahan, 2007). This window includes a period in mid-adolescence where socio-emotional rewards are salient and the impulse control system is not acting as forcefully as it does later in adolescence; as a result, at this age short-term mindsets may be relatively strong (Steinberg, 2008; Steinberg et al., 2008), especially in contexts of immediate rewards and around peers (Defoe et al., 2015). Later, impulse control may increasingly inhibit the pursuit of rewards from peers. Supporting this, neuroimaging studies show delayed maturational changes in brain structures underlying impulse control, compared to the socio-emotional reward system (Albert et al., 2013; Dumontheil, 2016). In conclusion, all these developments may gradually diminish the effect of UUS on short-term mindsets over the course of adolescence. Future research could further explore this age effect.

### Limitations and Future Research

Our study has four main limitations. First, longitudinal data does not permit drawing causal conclusions. Second, the time lags of two years between waves do not enable fine-grained tracking of temporal dynamics. Therefore, we cannot rule out that our observed effects may in part be due to changes in short-term mindsets preceding changes in UUS frequency (reverse causality). Third, although we involved several time-varying controls, there may be other unmeasured variables that influence the associations in our dataset (Collischon & Eberl, 2020). Fourth, we do not know whether our findings generalize to other populations. However, our fixed-effects models with longitudinal data of this representative adolescent cohort in Zurich may be considered the most rigorous methodological approach to address the problems of correlational data analyses (Weulen Kranenbarg et al., 2018).

This set of limitations applies also to other studies documenting effects of short-term mindsets on selection into UUS. These studies either did not control for prior UUS (Maimon & Browning, 2010), or short-term mindsets were no longer significantly associated with later UUS after controlling for prior UUS (Müller et al., 2013). Unlike previous studies reporting associations between short-term mindsets and UUS (Janssen et al., 2018; Qu et al., 2021), in our study, the temporal ordering of the variables aligned with our hypotheses. The findings of those previous studies have been interpreted as evidence for selection effects, but they may also (partially) reflect socialization effects. This raises the intriguing question of how future research may disentangle selection and socialization effects and their interactions.

Ideally, future research could collect more fine-grained data to track the temporal dynamics of the association of UUS and short-term mindsets. For example, people could be prompted via a smartphone application to provide data on these variables on a weekly basis over an extended period of time (i.e., experience sampling). Such data would enable us to analyze selection effects into UUS because of short-term mindsets, and the within-individual development of short-term mindsets in response to UUS. It may also help researchers estimate for how long increased short-term mindset levels are sustained after episodes of UUS. Lastly, collecting additional data about online communication and behavior may allow testing whether our findings also hold for the (sustained) impact of online socializing on short-term mindsets and online offending (e.g., cybercrime, cyberbullying).

Future research could also involve more encompassing measures of peer delinquency. Our measure focused on assault or shoplifting in the past year for a maximum of two nominated best friends. We used a proportion score to best reflect variety in the data. Nevertheless, more detailed data on friendship networks and peer’s delinquency could more accurately portray the relationships and potential deviant influence of peers. Data on the delinquency of peers present *during* UUS could particularly improve the investigation of interaction effects.

Future research could also include additional indicators of short-term mindsets. We found larger coefficients (effect sizes) for impulsivity and sensation seeking than for future orientation. However, we measured future orientation with a scale assessing only educational aspirations, that is, future school orientation. UUS was no longer significantly associated with this measure when all control variables, including parenting, were incorporated. Although we did not predict a weaker association between UUS and (low) future orientation, we interpret this finding as follows: UUS is a context that may reward impulsivity and sensation-seeking rather than punish educational aspirations. Future research may test this interpretation by including additional indicators of future orientation, beyond educational aspirations.

### Practical Implications

Our paper’s focus on negative outcomes of UUS that are associated with short-term mindsets—victimization, deviant behavior, and delinquency (Badura et al., 2018; Chen, 2009; Osgood et al., 1996)—does not imply that young people should not engage in UUS. Socializing with peers is key for socioemotional development and gaining independence in navigating the social environment (Blakemore & Mills, 2014; Little, 2020). How can we reduce undesirable correlates of UUS while also retaining its benefits?

First, it may help if adolescents who frequently spend time in UUS additionally also undertake some structured activities (with their peers) in their leisure time. Socializing has fewer negative consequences when accompanied by structure or supervision (e.g., sports; youth club). Structured youth activities can help decrease risky behavior, thus reducing the detrimental outcomes—and importantly, this seems to holds even for youth who do also frequently engage in UUS (Badura et al., 2018; Zill et al., 1995).

Second, we may attempt to promote future orientation among youth who frequently engage in UUS. Future orientation is associated with less delinquent behavior (Stoddard et al., 2015; van Gelder et al., 2018). Interventions that increase the vividness of one’s future self can reduce later deviance and delinquency (van Gelder et al., 2013; Gelder et al., 2015, 2022). Programs that reduce impulsive thinking among economically disadvantaged adolescents reduce delinquency (Heller et al., 2017). Such programs and interventions might mitigate short-term mindsets. Parents, practitioners, and social workers may invite youth who frequently engage in UUS to discuss pathways towards positive futures, rather than focusing conversations on judging current behaviors that challenge their futures (e.g., smoking, limited investment in education).

### Electronic Supplementary Material

Below is the link to the electronic supplementary material.

Supplementary Material 1

## Data Availability

Supplemental material can be found in the full text tab for this article. The data subset from the Zurich Project on the Social Development of Children and Youths (z-proso) that supports the findings of this study are openly available together with analysis syntaxes and metadata on DataVerseNL via 10.34894/Y90TQG. This is a preregistered study; the preregistration can be found via https://osf.io/5vzt4.

## Footnotes

1 We define *impulsivity* as a tendency to act on immediate urges (DeYoung & Rueter, 2016, p. 348), *sensation-seeking* as the tendency to accept risks in the pursuit of exciting behaviors despite potential future costs (Burt & Simons, 2013; Zuckerman, 1994), and *future orientation* as the tendency to make plans and set goals for the future, and to act in ways that align with these plans and goals (Corral-Verdugo & Pinheiro, 2006; Steinberg et al., 2009). Note that what we call sensation-seeking has also been referred to as ‘thrill seeking’(Burt & Simons, 2013), and ‘risk-seeking’ (Grasmick et al., 1993) or ‘risk-taking’ (Gottfredson & Hirschi, 1990, p. 90) in the criminological literature.

2 It is possible that adolescents learn only to embrace short-term mindsets in a similar context, that is, around peers (e.g., during UUS), and not in general. They may just “define [the behavior] as desirable or justified in a situation discriminative for the behavior” (Akers, 1998, p. 50). Nevertheless, the available evidence suggests that repeated exposure to situations in which peers model and encourage present prioritization can lead to the adoption of such preferences beyond the immediate context of being with peers, or socialization effects. If UUS contributes to short-term mindsets in such a way, this may explain non-situational effects of UUS on deviance.

3 This is consistent with research on decision making, which finds current behavior to often be the result of the experienced consequences of that behavior in the past (Anwar & Loughran, 2011; Burgess & Akers, 1966; Tittle et al., 2012). People update their preferences (e.g., the salience of immediate rewards compared to long-term costs) based on incoming information and responses to their actions, such as what behavior was rewarded (or punished) during UUS (Hoeben & Thomas, 2019; Rees & Winfree, 2017).

4 Study website: http://www.jacobscenter.uzh.ch/de/research/zproso.

5 The approval number for this project is #2018.2.12.

6 https://osf.io/5vzt4.

7 10.34894/Y90TQG.

8 We computed the mean of four items at wave 8 as this wave does not include the item “Meet up with friends at a house without adults”.

9 Item response theory assigns a person the *θ* level with the maximum likelihood to reproduce the pattern of responses on the items (Osgood et al., 2002; Thomas, 2019). With one parameter per item, Rasch models permit different base rates across items (item difficulty). In addition, Rasch models assume the same discrimination or validity for each item (Andrich, 1988). Two-parameter models do not require this assumption but instead introduce an additional discrimination parameter to model items’ unique ability to differentiate on the latent dimension (Osgood et al., 2002). A likelihood-ratio test (Cohen et al., 1996) recommended the use of two-parameter models for all waves except for wave 4, for which the test was just not significant (*p* = .055). For the comparability of the theta levels we therefore opted to use two-parameter models for all waves. These models estimate “the probability that individual *j* endorses item *i* as a function of *j*’s underlying ability (*θ*_*j*_), the difficulty of the question (*b*_*i*_), and how well the item discriminates between individuals of different levels of ability (*a*_*i*_)” (Thomas, 2019, p. 11).

10 Our results did not change if we instead used an graded response model on an ordered count measure for these offending items (correlations between these two measures of offending was *r* = .988).

11 At wave 4, the data set involves only two items each for parental supervision and for adolescent disclosure. We calculated the mean of these available items. Wave 8 includes neither subscales of parental monitoring.

12 Wave 8 did not include the measure of parental involvement.
