# Supplementary material for: Beyond the Situation: Hanging Out with Peers now is Associated with Short-Term Mindsets Later
Source: J Dev Life Course Criminol. 2024 Feb 17;10(1):51–72. doi: 10.1007/s40865-024-00249-2 (PMC11147868; doi:10.1007/s40865-024-00249-2)
Supplement: Supplementary file 1 — Supplementary Material 1 [file 40865_2024_249_MOESM1_ESM.docx]

SUPPLEMENTARY MATERIALS FOR

**Beyond the situation: Hanging out with peers now is associated with short-term mindsets later** ^[[1]](#footnote-1)^*

SEBASTIAN L. KÜBEL, JESSICA R. DEITZER, WILLEM E. FRANKENHUIS, DENIS RIBEAUD, MANUEL P. EISNER, and JEAN-LOUIS VAN GELDER

**APPENDIX A. SPECIFICATION OF VARIABLES**

***Independent variable:*** *Unstructured Unsupervised Socializing (UUS)*

Unstructured unsupervised socializing was computed as the mean of the following five items: What do you do in your free time when you are not at home? How often do you do the following things? (Rated on a six-point Likert scale from 1 = never to 6 = (almost) every day)

- Meet up with friends in the evening and do something with them.*
- Meet up with friends at a house without parents. **
- Go to a party or festival without parents, in the evening. ***
- Hang around with friends in a park, in the train station, or in a shopping mall, and have fun, in the afternoon.
- Hang around with friends in a park, in the train station, or in a shopping mall, and have fun, in the evening.

There were small differences in two items over the waves: * in wave 4, the time was specified and read as “on Friday or Saturday evenings”; ** this item was not available in wave 8; *** in waves 6-8, the phrase “without parents” was excluded and replaced by “with friends.”

***Dependent variable:*** *Short-term mindsets (the same as control of prior short-term mindsets)*

For the short-term mindsets variables of impulsivity, sensation-seeking, and future orientation, we computed the mean of the scales, respectively.

All items were scored on a four-point Likert scale ranging from 1 = false to 4 = true.

*Impulsivity:*

- I often act on the spur of the moment without stopping to think.
- I often do whatever brings me pleasure here and now, even at the cost of some distant goal.

*Sensation-Seeking*

- Sometimes I take risks just for the fun of it.
- Excitement and adventure are more important to me than security.

*Future Orientation:*

- I try hard at school to have a good job later in life.
- When I grow up I want to have an interesting job, and I’m doing everything now to work towards that goal.
- Doing well at school is very important to me.

***Control variables:***

*Offending*

Offending is positively associated with UUS (Hoeben & Weerman, 2016; Maimon & Browning, 2010) and short-term mindsets (Forrest et al., 2019; van Gelder et al., 2018, 2020).

Fourteen items refer to prevalence of the following types of offending in the past twelve months (all items scored dichotomously: 0 = never, 1= at least once). We derive individual values (theta levels *θ*) on a latent trait dimension (criminal propensity) by applying item response theory on these dichotomous items.

Note that the items driving w/o license, burglary/steal from car, drug dealing, graffitiing, carry a weapon, threat/extortion, and robbery were not available in wave 4.

*Offending questionnaire:* In the past 12 months, have you ever…? (0 = no, 1 = yes)

- Steal at school: … stolen something at school?
- Steal at home: … stolen something at home?
- Shoplifting < $50: … stolen something from a shop or kiosk that is worth less than 50 CHF? (approx. 50$)
- Shoplifting > $50: … stolen something from a shop or kiosk that is worth more than 50 CHF?
- Vehicle theft: … stolen a bicycle or another vehicle?
- Driving w/o license: … driven a motor vehicle (car, motorbike) without having a valid driving license?
- Burglary / steal from car: … broken into a car or a building (e.g. house, shop) to steal something from there?
- Drug dealing: … sold drugs (e.g. hashish, cocaine, ecstasy)?
- Graffitiing: … sprayed graffiti on buildings or on public transport, or made “tags“?
- Vandalism: … purposely damaged windows, street lamps, seats on the tram, train, or bus, or other similar things?
- Carry a weapon: … carried a weapon or other dangerous object to protect yourself or to threaten others or attack them?
- Threat/Extortion: … threatened anyone with violence to obtain money or things?
- Robbery: … forcibly taken money or things from someone?
- Assault: … purposely hit, kicked, or cut someone, and injured him or her in the process?

*Affiliations with delinquent peers*

Delinquent peers are positively linked to UUS (Archer et al., 2022; Hoeben & Weerman, 2016). Affiliation with delinquent peers is also associated with decreased self-control (Burt et al., 2006; Jennings et al., 2013; Meldrum et al., 2012).

As a measure of affiliation with delinquent peers, we computed the proportion score of two nominated friends that engaged in either assault and/or shoplifting in the past year (0 = neither best friend has done either delinquent act or sole nominated best friend has not done either delinquent act, 0.5 = only one of two nominated best friends has done either delinquent act; 1 = both best friends or sole nominated best friend has done either delinquent act; coded as missing if no best friend was nominated).

Referring to the two best friends denominated by the participant (scored yes / no):

- In the last year, has he/she purposely hit or kicked another adolescent and injured them in the process?
- In the last year, has he/she stolen something from a shop, kiosk, or shopping mall?

*Parental monitoring*

Parental monitoring is associated with less time spent in UUS (Archer et al., 2022; Janssen et al., 2014, 2016, 2018) and with higher levels of self-control (Janssen et al., 2016, 2018; Meldrum, 2008).

Parental monitoring was measured with two subscales; the mean of the following four parental supervision items, and the mean of the inverted scores of three adolescent disclosure items presented below.

All items were scored on a four-point Likert scale from 1 = never to 4 = often/always.

*Parental supervision subscale*

- When you go out in your free time, your parents ask you where you are going.
- When you go out in your free time your parents tell you what time you have to be home by.
- You have to tell your parents who you meet with in your free time.
- Your parents ask you what you get up to in your free time.

*Adolescent disclosure subscale*

- You keep secret from your parents what you do in the evenings and at the weekends.
- You leave your house without telling your parents where you are going.
- You stay out in the evening past the time you are supposed to be home.

*Parental involvement*

Parental involvement is negatively associated with both UUS (Janssen et al., 2014, 2018) and low self-control (Janssen et al., 2016, 2018; Nie et al., 2016).

Parental involvement was calculated as the mean of the following six items: Please mark with crosses which of the things below occur never, rarely, sometimes or often in your home.

All items were scored on a four-point Likert scale from 1 = never to 4 = often/always.

- Your parents talk to you about your friends or about the other students in your class.
- You play games or do other fun things with your parents.
- Your parents help you when you struggle with your homework.
- Your mother or father hugs you to comfort you when you are sad.
- Your parents are interested in what you do.
- When you have a problem, you can talk to your parents about it.

*Age*

Age was measured in years, with two decimals.

**APPENDIX B. INTERNAL CONSISTENCIES.**

Table B. Internal consistencies of the scales at each wave.

|  | measure^1^ | wave 4 | wave 5 | wave 6 | wave 7 | wave 8 |
| --- | --- | --- | --- | --- | --- | --- |
| UUS | Cronbach’s *α* | 0.77 | 0.80 | 0.79 | 0.77 | 0.69^2^ |
| impulsivity | MIIC | 0.26 | 0.27 | 0.28 | 0.33 | 0.33 |
| sensation-seeking | MIIC | 0.43 | 0.55 | 0.54 | 0.58 | 0.55 |
| future orientation | MIIC | --- | 0.48 | 0.52 | 0.49 | --- |
| parental supervision | MIIC | 0.32^3^ |  |  |  | --- |
|  | Cronbach’s *α* |  | 0.70 | 0.72 | 0.70 |  |
| adolescent disclosure | MIIC | 0.30 | 0.37 | 0.32 | 0.30 | --- |
| parental involvement | Cronbach’s *α* | 0.66 | 0.75 | 0.77 | 0.77 | --- |

Cronbach’s alpha is a biased estimate for scales with few items. The (mean) Spearman-Brown inter-item correlation (MIIC) is a more appropriate measure for internal consistency (Eisinga et al., 2013). We therefore use the MIIC for scales with up to three items, and Cronbach’s *α* for scales with four or more items.

2 Wave 8 does not include the item “Meet up with friends at a house without adults”. Thus, there are only four UUS items in wave 8.

3 At wave 4, the data set involves only two items for parental supervision, and two items for adolescent disclosure. We calculated the mean of these available items for wave 4 and provide the MIIC as measure for internal reliability. Wave 8 includes neither subscales of parental monitoring.

**APPENDIX C. DESCRIPTIVE STATISTICS.**

Table C. Descriptive statistics of the study variables over the waves.

|  | wave | *n* | *% of N at wave* | *M* (SD) |
| --- | --- | --- | --- | --- |
| UUS | wave 4 | 1,120 | 97.56 | 2.14 (1.00) |
|  | wave 5 | 1,360 | 99.56 | 2.76 (1.05) |
|  | wave 6 | 1,444 | 99.79 | 2.99 (1.00) |
|  | wave 7 | 1,303 | 99.77 | 2.98 (0.92) |
|  | wave 8 | 1,179 | 99.92 | 2.95 (0.87) |
| impulsivity | wave 4 | 1,145 | 99.74 | 1.98 (0.68) |
|  | wave 5 | 1,360 | 99.56 | 2.30 (0.62) |
|  | wave 6 | 1,444 | 99.79 | 2.37 (0.57) |
|  | wave 7 | 1,294 | 99.08 | 2.34 (0.60) |
|  | wave 8 | 1,179 | 99.92 | 2.19 (0.61) |
| sensation-seeking | wave 4 | 1,145 | 99.74 | 1.85 (0.78) |
|  | wave 5 | 1,358 | 99.41 | 2.12 (0.77) |
|  | wave 6 | 1,444 | 99.79 | 2.20 (0.73) |
|  | wave 7 | 1,294 | 99.08 | 2.13 (0.74) |
|  | wave 8 | 1,179 | 99.92 | 2.00 (0.70) |
| future orientation | wave 5 | 1,360 | 99.56 | 3.23 (0.61) |
|  | wave 6 | 1,445 | 99.86 | 3.16 (0.61) |
|  | wave 7 | 1,196 | 91.58 | 3.16 (0.60) |
| criminal propensity | wave 4 | 1,147 | 99.91 | 0.00 (0.61) |
|  | wave 5 | 1,359 | 99.49 | 0.00 (0.74) |
|  | wave 6 | 1,445 | 99.86 | 0.00 (0.74) |
|  | wave 7 | 1,301 | 99.62 | 0.00 (0.73) |
|  | wave 8 | 1,179 | 99.92 | 0.00 (0.68) |
| delinquent peer affiliations | wave 4 | 1,059 | 92.25 | 0.18 (0.33) |
|  | wave 5 | 1,270 | 92.97 | 0.17 (0.32) |
|  | wave 6 | 1,393 | 96.27 | 0.18 (0.32) |
|  | wave 7 | 1,158 | 88.67 | 0.14 (0.30) |
|  | wave 8 | 1,033 | 87.54 | 0.10 (0.26) |
| age | wave 4 | 1,143 | 99.56 | 11.33 (0.37) |
|  | wave 5 | 1,365 | 99.93 | 13.67 (0.36) |
|  | wave 6 | 1,446 | 99.93 | 15.44 (0.36) |
|  | wave 7 | 1,306 | 100.00 | 17.45 (0.37) |
|  | wave 8 | 1,180 | 100.00 | 20.58 (0.38) |
| parental supervision | wave 4 | 1,147 | 99.91 | 3.63 (0.54) |
|  | wave 5 | 1,361 | 99.63 | 3.09 (0.64) |
|  | wave 6 | 1,446 | 99.93 | 3.17 (0.63) |
|  | wave 7 | 1,301 | 99.62 | 3.00 (0.67) |
| adolescent disclosure | wave 4 | 1,147 | 99.91 | 3.43 (0.59) |
|  | wave 5 | 1,362 | 99.71 | 3.13 (0.64) |
|  | wave 6 | 1,446 | 99.93 | 3.17 (0.63) |
|  | wave 7 | 1,301 | 99.62 | 2.92 (0.70) |
| parental involvement | wave 4 | 1,147 | 99.91 | 3.34 (0.46) |
|  | wave 5 | 1,362 | 99.71 | 3.08 (0.59) |
|  | wave 6 | 1,446 | 99.93 | 3.01 (0.62) |
|  | wave 7 | 1,301 | 99.62 | 2.96 (0.63) |

**APPENDIX D. CORRELATIONS.**

Table D. Spearman correlations between mean levels of predictors and mean levels of short-term mindsets indicators across all waves.

|  | *M* (*SD*) | *n* | UUS | criminal  propensity | delinquent  peers | parental  supervision | adolescent  disclosure | parental  involvement | age |
| --- | --- | --- | --- | --- | --- | --- | --- | --- | --- |
| wave 4 impulsivity | 1.98  (0.68) | 1,145 | .197*** | .209*** | .165*** | -.137*** | -.247*** | -.078** | .029 |
| wave 5 impulsivity | 2.30  (0.62) | 1,360 | .278*** | .240*** | .156*** | -.088** | -.308*** | -.153*** | .019 |
| wave 6 impulsivity | 2.37  (0.57) | 1,444 | .211*** | .224*** | .100*** | -.109*** | -.237*** | -.108*** | .005 |
| wave 7  impulsivity | 2.34  (0.60) | 1,294 | .267*** | .196*** | .116*** | -.143*** | -.239*** | -.136*** | .018 |
| wave 8  impulsivity | 2.19  (0.61) | 1,179 | .195*** | .142*** | .081** | --- | --- | --- | .011 |
| wave 4  sensation-seeking | 1.85  (0.78) | 1,145 | .288*** | .306*** | .228*** | -.188*** | -.324*** | -.135*** | .084** |
| wave 5 sensation-seeking | 2.12  (0.77) | 1,358 | .362*** | .392*** | .289*** | -.142*** | -.395*** | -.181*** | .021 |
| wave 6 sensation-seeking | 2.20  (0.73) | 1,444 | .323*** | .363*** | .244*** | -.171*** | -.387*** | -.199*** | -.005 |
| wave 7 sensation-seeking | 2.13  (0.74) | 1,294 | .368*** | .379*** | .269*** | -.153*** | -.362*** | -.173*** | -.013 |
| wave 8  sensation-seeking | 2.00  (0.70) | 1,179 | .264*** | .359*** | .181*** | --- | --- | --- | .009 |
| wave 5  future orientation | 3.23  (0.61) | 1,360 | -.164*** | -.249*** | -.168*** | .145*** | .296*** | .230*** | -.034 |
| wave 6  future orientation | 3.16  (0.61) | 1,445 | -.133*** | -.244*** | -.171*** | .148*** | .263*** | .206*** | .004 |
| wave 7  future orientation | 3.16  (0.60) | 1,196 | -.193*** | -.233*** | -.183*** | .148*** | .278*** | .233*** | .010 |

Note. Each of the column headers refers to the same wave as indicated in the row headers. The parenting variables are not available in wave 8 (parental supervision; adolescent disclosure; parental involvement).

* *p* < .05; ** *p* < .01; ****p*<.001.

**APPENDIX E. EXPLORATORY ANALYSES INCLUDING THE INTERACTION OF DELINQUENT FRIENDS AND UUS.**

Table E1. Results of the fixed-effects models including the interaction of delinquent peers and UUS, but no control variables.

|  | impulsivity | sensation-seeking | future orientation |
| --- | --- | --- | --- |
|  | *b* (SE) | *b* (SE) | *b* (SE) |
| 1. **Model parameters** | | | |
| UUS | 0.093*** (0.012) | 0.150*** (0.013) | -0.059*** (0.016) |
| delinquent peers | 0.101** (0.036) | 0.231*** (0.037) | -0.144** (0.042) |
| UUS x delinquent peers | -0.007 (0.034) | 0.003 (0.035) | 0.059 (0.039) |
| constant | 2.246*** (0.002) | 2.066*** (0.002) | 3.189*** (0.003) |
| 1. **Model characteristics** | | | |
| *n* (unique individuals) | 1,516 | 1,516 | 1,467 |
| *N* (person-waves) | 5,887 | 5,886 | 3,727 |
| rho | .370 | .479 | .534 |
| model | *F*(3,1515) = 23.47*** | *F*(3,1515) = 63.20*** | *F*(3,1466) = 9.64*** |

Note. * *p* < .05; ** *p* < .01; ****p* < .001. UUS = unstructured unsupervised socializing. *b* = unstandardized coefficient. SE = robust Huber/White/sandwich standard error. rho = fraction of variance due to fixed effects. The model *F*-statistic tests whether all fixed effects are zero.

Table E2. Results of the fixed-effects models including the interaction off delinquent peers and UUS and the control variables offending and age.

|  | impulsivity | sensation-seeking | future orientation |
| --- | --- | --- | --- |
|  | *b* (SE) | *b* (SE) | *b* (SE) |
| 1. **Model parameters** | | | |
| UUS | 0.081*** (0.012) | 0.129*** (0.013) | -0.044** (0.016) |
| delinquent peers | 0.090* (0.036) | 0.179*** (0.037) | -0.105* (0.042) |
| UUS x delinquent peers | -0.015 (0.033) | -0.009 (0.035) | 0.057 (0.039) |
| *control variables:* | | | |
| age | 0.016*** (0.003) | 0.012*** (0.003) | -0.023*** (0.005) |
| offending | 0.071*** (0.018) | 0.157*** (0.018) | -0.104*** (0.021) |
| constant | -1.997*** (0.045) | -1.888*** (0.044) | 3.544*** (0.085) |
| 1. **Model characteristics** | | | |
| *n* (unique individuals) | 1,516 | 1,516 | 1,467 |
| *N* (person-waves) | 5,872 | 5,871 | 3,720 |
| rho | .363 | .459 | .529 |
| model | *F*(5,1515) = 25.43*** | *F*(5,1515) = 57.08*** | *F*(5,1466) = 15.83*** |

Note. * *p* < .05; ** *p* < .01; ****p* < .001. UUS = unstructured unsupervised socializing. *b* = unstandardized coefficient. SE = robust Huber/White/sandwich standard error. rho = fraction of variance due to fixed effects. The model *F*-statistic tests whether all fixed effects are zero.

Table E3. Results of the fixed-effects models including the interaction off delinquent peers and UUS; including all control variables.

|  | impulsivity | sensation-seeking | future orientation |
| --- | --- | --- | --- |
|  | *b* (SE) | *b* (SE) | *b* (SE) |
| 1. **Model parameters** | | | |
| UUS | 0.068*** (0.015) | 0.105*** (0.015) | -0.022 (0.016) |
| delinquent peers | 0.066† (0.039) | 0.153*** (0.041) | -0.079† (0.042) |
| UUS x delinquent peers | -0.015 (0.038) | -0.015 (0.039) | 0.063† (0.038) |
| *control variables:* | | | |
| age | 0.039*** (0.005) | -0.021*** (0.005) | -0.012* (0.006) |
| offending | 0.043* (0.020) | 0.115*** (0.022) | -0.085*** (0.020) |
| parental supervision | -0.057** (0.020) | -0.017 (0.022) | 0.016 (0.022) |
| adolescent disclosure | -0.110*** (0.022) | -0.170*** (0.021) | 0.096*** (0.022) |
| parental involvement | -0.043 (0.027) | -0.150*** (0.026) | 0.151*** (0.027) |
| constant | -2.348*** (0.145) | -2.833*** (0.151) | 2.573*** (0.159) |
| 1. **Model characteristics** | | | |
| *n* (unique individuals) | 1,514 | 1,514 | 1,467 |
| *N* (person-waves) | 4,834 | 4,833 | 3,714 |
| rho | .383 | .474 | .518 |
| model | *F*(8,1513) = 38.46*** | *F*(8,1513) = 50.55*** | *F*(8,1466) = 18.53*** |

Note. The model with all controls involves waves 4-7. † *p* < .10; * *p* < .05; ** *p* < .01; ****p* < .001. UUS = unstructured unsupervised socializing. *b* = unstandardized coefficient. SE = robust Huber/White/sandwich standard error. rho = fraction of variance due to fixed effects. The model *F*-statistic tests whether all fixed effects are zero.

**APPENDIX F. EXPLORATORY ANALYSES WITH SHORT-TERM MINDSETS FACTOR.**

Table F. Results of the fixed-effects models with short-term mindsets measured as a factor

|  | bivariate model | controls except parenting | all controls | |
| --- | --- | --- | --- | --- |
|  | *b* (SE) | *b* (SE) | *b* (SE) | |
| 1. **Model parameters** | | | |  |
| UUS | 0.153*** (0.013) | 0.137*** (0.014) | 0.114*** (0.017) |  |
| *control variables:* | | | |  |
| age |  | -0.009* (0.003) | -0.039*** (0.006) |  |
| offending |  | 0.189*** (0.021) | 0.150*** (0.023) |  |
| parental supervision |  |  | -0.018 (0.024) |  |
| adolescent disclosure |  |  | -0.175*** (0.024) |  |
| parental involvement |  |  | -0.199*** (0.031) |  |
| delinquent peers |  | 0.182*** (0.041) | 0.145** (0.044) |  |
| constant | -0.426*** (0.035) | -0.278*** (0.053) | 1.445*** (0.177) |  |
| 1. **Model characteristics** | | | |  |
| *n* (unique individuals) | 1,519 | 1,514 | 1,511 | |
| *N* (person-waves) | 6,165 | 5,690 | 4,653 | |
| rho | .477 | .453 | .473 | |
| model | *F*(1,1518) = 147.91*** | *F*(4,1513) = 66.76*** | *F*(7,1510) = 51.74*** | |

Note. The model with all controls involves waves 4-7. † *p* < .10; * *p* < .05; ** *p* < .01; ****p* < .001. UUS = unstructured unsupervised socializing. *b* = unstandardized coefficient. SE = robust Huber/White/sandwich standard error. rho = fraction of variance due to fixed effects. The model *F*-statistic tests whether all fixed effects are zero.

1. * Published 2024 in *Journal of Developmental and Life-Course Criminology*,

   https://doi.org/10.1007/s40865-024-00249-2 [↑](#footnote-ref-1)
